# Combining Freestanding Ferroelectric Perovskite Oxides with Two-Dimensional Semiconductors for High Performance Transistors

**DOI:** 10.1021/acs.nanolett.2c02395

**Published:** 2022-09-15

**Authors:** Sergio Puebla, Thomas Pucher, Victor Rouco, Gabriel Sanchez-Santolino, Yong Xie, Victor Zamora, Fabian A. Cuellar, Federico J. Mompean, Carlos Leon, Joshua O. Island, Mar Garcia-Hernandez, Jacobo Santamaria, Carmen Munuera, Andres Castellanos-Gomez

**Affiliations:** † Materials Science Factory, Instituto de Ciencia de Materiales de Madrid (ICMM-CSIC), Madrid E-28049, Spain; ‡ GFMC, Department Fisica de Materiales, Facultad de Fisica, Universidad Complutense 28040 Madrid, Spain; § Laboratorio de Heteroestructuras con aplicación en spintrónica, Unidad Asociada UCM/CSIC, 28040 Madrid, Spain; ∥ Instituto Pluridisciplinar, Universidad Complutense de Madrid, 28040 Madrid, Spain; ⊥ School of Advanced Materials and Nanotechnology, Xidian University, Xi’an 710071, China; # Department of Physics and Astronomy, University of Nevada Las Vegas, Las Vegas, Nevada 89154, United States

**Keywords:** freestanding complex oxide, ferroelectric perovskite oxide, ferroelectric field effect transistor, molybdenum disulfide (MoS_2_), barium titanate (BaTiO_3_)

## Abstract

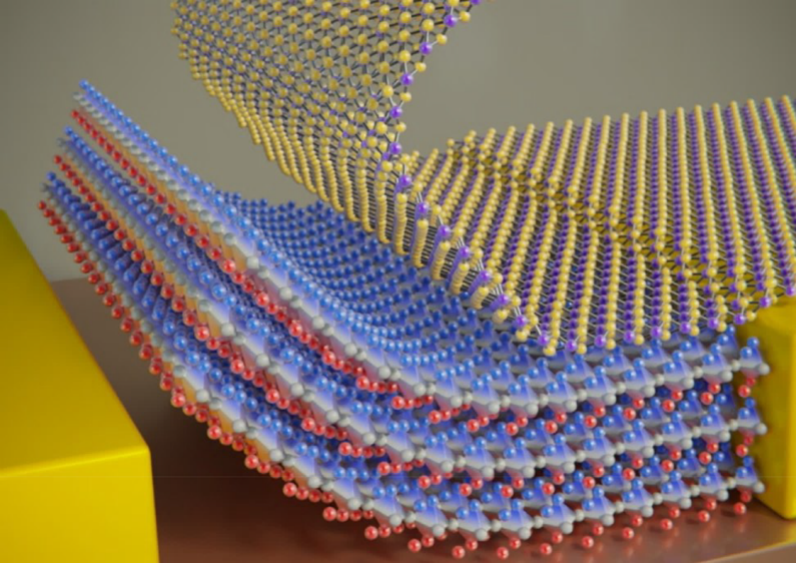

We demonstrate the fabrication of field-effect transistors based on single-layer MoS_2_ and a thin layer of BaTiO_3_ (BTO) dielectric, isolated from its parent epitaxial template substrate. Thin BTO provides an ultrahigh-κ gate dielectric effectively screening Coulomb scattering centers. These devices show mobilities substantially larger than those obtained with standard SiO_2_ dielectrics and comparable with values obtained with hexagonal boron nitride, a dielectric employed for fabrication of high-performance two-dimensional (2D) based devices. Moreover, the ferroelectric character of BTO induces a robust hysteresis of the current vs gate voltage characteristics, attributed to its polarization switching. This hysteresis is strongly suppressed when the device is warmed up above the tetragonal-to-cubic transition temperature of BTO that leads to a ferroelectric-to-paraelectric transition. This hysteretic behavior is attractive for applications in memory storage devices. Our results open the door to the integration of a large family of complex oxides exhibiting strongly correlated physics in 2D-based devices.

The development of methods and tools to deterministically transfer two-dimensional (2D) materials in 2010^1−3^ opened the door to fabricate artificial matter by simply stacking 2D materials on top of each other, without being constrained by the rigid limitations of epitaxial growth: lattice parameter matching, complexity of the technique, and price of the required tools.^4^ Since then, this emerging field of research, the so-called van der Waals heterostructures,^5−9^ has continued to grow at a tremendous pace. Novel physical phenomena^10^ and device concepts^11−13^ have been achieved by these straightforward methods of combining 2D materials.

Apart from vertically stacking 2D materials, a great deal of interest has been focused on using these deterministic transfer methods to combine/integrate 2D materials with other families or classes of materials, including epitaxially grown complex oxides, in mixed-dimensional van der Waals heterostructures.^14−16^ Within this context, the isolation of 2D non-van der Waals materials opens up an interesting avenue.^17−21^ In particular, the recent fabrication of freestanding layers of complex transition metal oxides of just a few unit cells in thickness^22−28^ provides a new research arena as this family of materials presents a whole plethora of strongly correlated physics that is scarcely present in only a handful of van der Waals 2D materials. The integration of these freestanding complex oxides with 2D materials, however, was not reported until very recently by two research groups in parallel with preparation of our work. In these two recent works^29,30^ SrTiO_3_ (STO) thin layers are isolated freestanding and used as high-κ dielectrics for field-effect transistors based on 2D materials. Yang et al. show that in devices utilizing a freestanding layer of STO, ON/OFF ratios of 10^8^ and subthreshold swings of 66 mV/dec can be achieved.^30^ Huang et al. show that the STO layer is exceptional for use as a dielectric layer because it possesses a subone-nanometer capacitance equivalent thickness with a low leakage current (less than 10^–2^ A·cm^–2^ at 2.5 MV·cm^–1^).^29^

Prior to the isolation of freestanding layers of complex oxides, 2D materials were already integrated with complex oxide dielectrics epitaxially grown onto 3D oxide substrates to improve and add new functionalities to 2D-based devices.^31^ These previous works show a glimpse of the potential of combining van der Waals 2D materials with freestanding complex oxides. Particularly appealing in this field is the use of ferroelectric oxides, presenting a spontaneous polarization that can be reversed by a suitable electric field in the opposite direction.^32^ This polarization influences the electronic properties of the 2D material-oxide interface, and its switchable character provides an additional tuning parameter for device operation. Memristive properties have, in this way, been added to 2D-based field effect transistors and tunnel junctions.^33−42^ As already achieved with the dielectric STO,^29,30^ any step forward in facilitating the stackable aspect of these ferroelectric components would immediately expand their potential applications, using the van der Waals integration strategy to fabricate novel hybrid 2D heterostructures.

In this work, we isolate freestanding crystalline thin layers of BTO, one of the most widely researched ferroelectric materials, and integrate them for use as a ferroelectric gate dielectric in field effect transistors based on single-layer MoS_2_ semiconducting channels. We found that BTO acts as a very effective high-κ dielectric, screening out Coulomb scattering centers and yielding larger mobility values than those obtained with standard SiO_2_ dielectric.^43^ In comparison with the use of 2D ferroelectric van der Waals materials such as CuInP_2_S_6_ as a dielectric layer,^44^ BTO offers a much higher ferroelectric moment^45^ (26 μC/cm^2^ vs 3.8 μC/cm^2^), electric permittivity^46^ (∼4000 vs ∼50) at room temperature, larger band gap^47−49^ (∼3.3 eV vs 2.6 eV) and higher Curie temperature^45^ (420 K vs 320 K). Moreover, the current versus gate voltage traces of the MoS_2_ field-effect transistors based on BTO dielectric present a very robust hysteresis, characteristic of the ferroelectric nature of BTO. This adds an additional characteristic beyond exceptional device properties when compared with recent results utilizing STO.^29,30^ This hysteretic behavior can be used to fabricate low energy consumption transistors or memory storage devices. We compare the performance of the fabricated BTO-based devices with that obtained in MoS_2_ transistors fabricated with hexagonal boron nitride (hBN) flakes of similar thickness finding comparable mobility values, indicating that the ultrahigh dielectric constant of BTO effectively screens out the charge impurities.

The BTO layers, 15–50 nm thick, were epitaxially grown by high pressure (pure oxygen) sputtering on (001) STO substrates covered by (15 nm) La_0.7_Sr_0.3_MnO_3_ (LSMO) epitaxial buffer layers. The LSMO layer is used as a sacrificial layer to enable the BTO delamination. We take advantage of the fact that a HCl and KI based solution etches LSMO very effectively without damaging the BTO film to release the BTO from the parent substrate (see Materials and Methods). Prior to the LSMO etching, a Gel-Film square (Gel-Film WF 4× 6.0 mil by Gel-Pac) is adhered to the BTO surface to facilitate the handling of the BTO once it detaches from the parent substrate. Figure 1a illustrates the steps employed to delaminate the BTO layer from its parent substrate and Figure 1b shows an optical image of a Gel-Film with a ∼2 by 2 mm^2^ BTO layer (25 nm thick), obtained after the release process.

**Figure 1:**
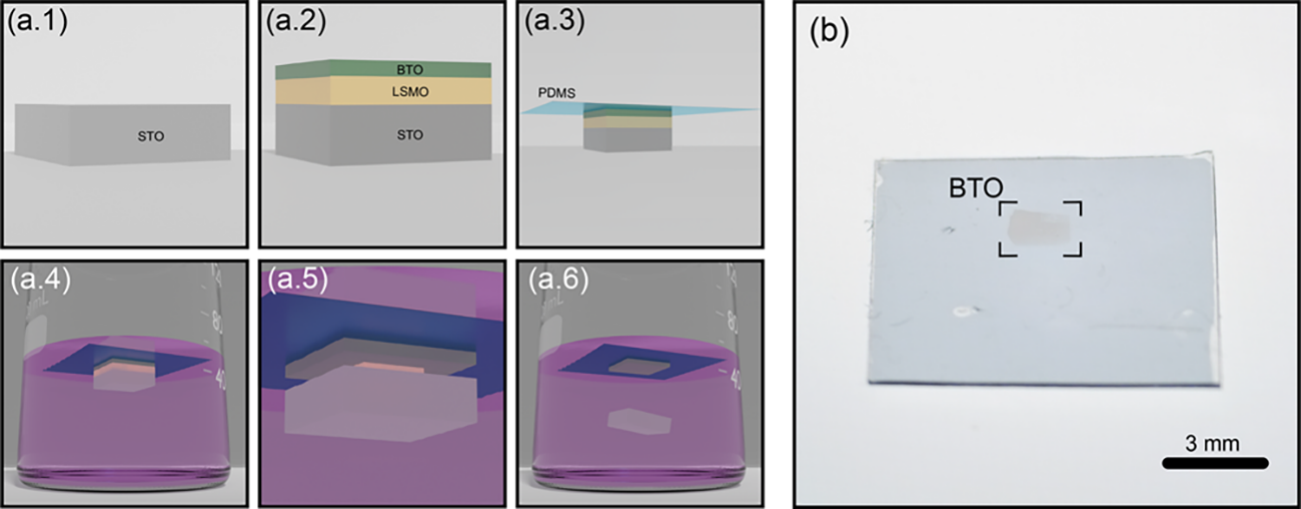
Isolation of freestanding BTO thin films. (a) Steps for the isolation of freestanding BTO films. A thin film of LSMO (15 nm) is epitaxially grown onto a STO substrate and subsequently a 15–50 nm BTO film is epitaxially grown on top. Then a Gel-Film substrate is adhered to the BTO surface and the stack is immersed in an etching solution (0.5 mL HCl (37%), 0.5 mL KI (3M), 10 mL H_2_O) that attacks selectively the LSMO and releases the BTO film. (b) Picture of a Gel-Film carrier substrate with a macroscopic film of BTO (25 nm thick) on its surface.

The structure and composition of as-isolated BTO freestanding layers were characterized by high-resolution scanning transmission electron microscopy and electron energy loss spectroscopy (STEM-EELS). Figure 2a shows a high-angle annular dark-field (HAADF) image of an as-isolated freestanding BTO layer released from the substrate and transferred onto a standard Si_3_N_4_ holey membrane. Details about sample preparation can be found in the Materials and Methods. Atomic resolution HAADF characterization of the same flake along the [001] direction evidence a highly ordered perovskite structure, as shown in the image of Figure 2b. Occasionally, stacking faults or other growth defects were observed in the images. Quantitative analysis of high-resolution STEM images (see the Supporting Information Figure S1) allows for the determination of the in-plane lattice parameters of the freestanding BTO layer. We obtain 4.10 and 4.13 Å for the in-plane lattice parameters along the high symmetry (*x* and *y*) directions of the tetragonal ferroelectric structure. These values are somewhat larger than the expected bulk in-plane values (4 Å) which could result from an in-plane polarization in the freestanding BTO layers. EELS spectra at the Ti-*L*_2,3_ and O-*K* edges were measured over the region shown in Figure 2b (see Figure 2c). Ti-*L*_*2,3*_ edge fine structure shows a clear splitting between t_2g_ and e_g_ peaks, characteristic of a 4+ Ti oxidation state, as expected from the BTO bulk stoichiometry. Quantification of the Ti oxidation state was carried out using a multiple linear least-squares fitting (MLLS) procedure to LaTiO_3_ (Ti ^3+^) and BaTiO_3_ bulk (Ti ^4+^) reference spectra and averaged values of 3.99 ± 0.01 were obtained.^50^ We additionally characterized the freestanding BTO flakes using optical microscopy, microreflectance, Raman spectroscopy, photoluminescence spectroscopy (see Supporting Information Figures S2, S3 and S4). Furthermore, X-ray diffraction and reflectivity has been used to determine the out-of-plane lattice parameter of the freestanding BTO films (finding a value of *c* = 4.00 Å, indicating relaxation of the crystal lattice as compared to the epitaxial BTO layers), see Supporting Information Figures S5 and S6.

**Figure 2:**
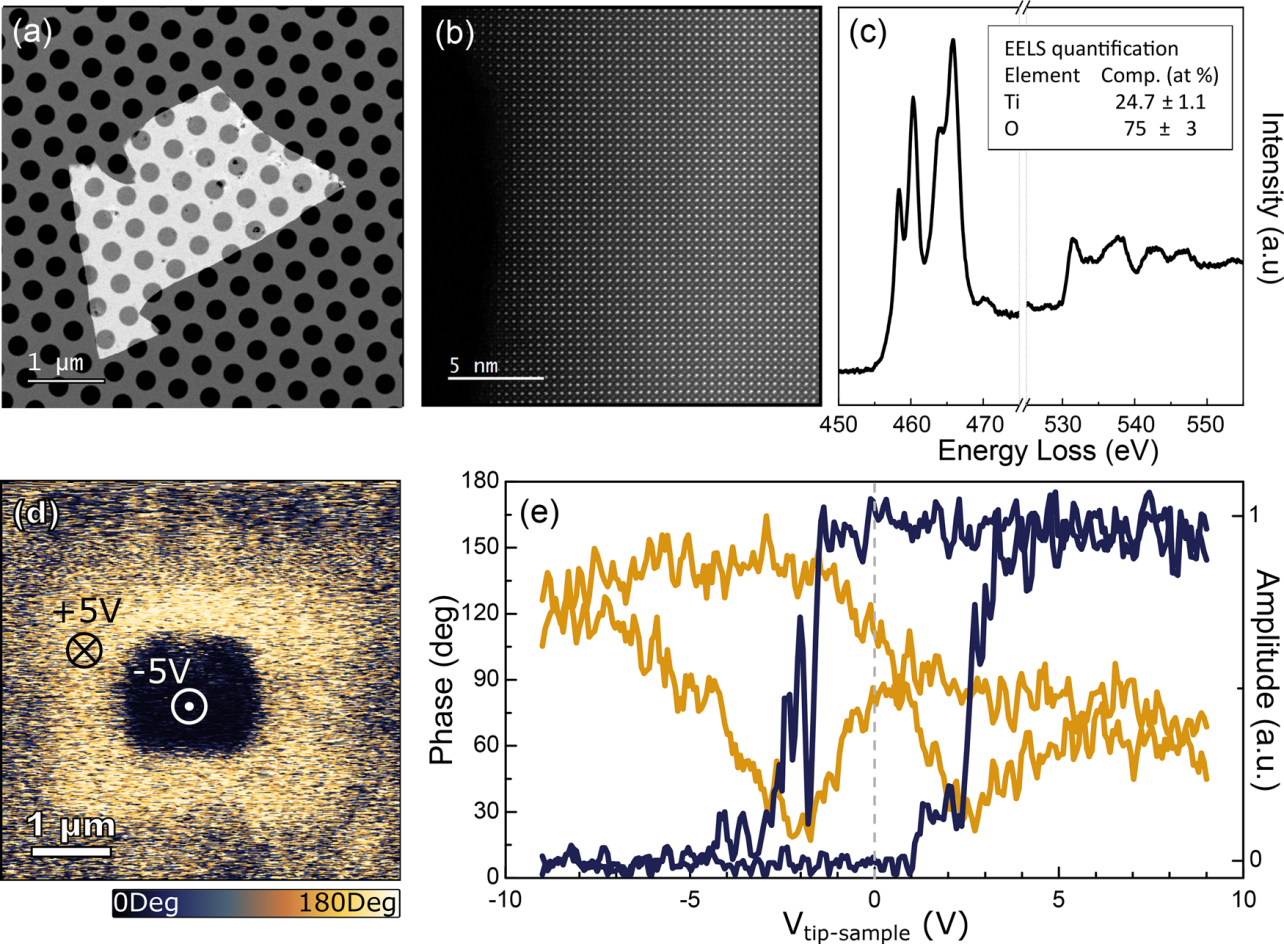
Structural, chemical, and ferroelectric characterization of freestanding BTO thin films. (a) Z-contrast scanning transmission electron microscopy (STEM) low-magnification image of a 30 nm freestanding BTO flake transferred over a holey Si_3_N_4_ membrane. (b) Atomic resolution image showing the atomically sharp edge of the BTO flake viewed along the [001] direction. (c) Electron energy loss spectra of the Ti*L*_*2,3*_ and O*K* edges acquired over the freestanding BTO flake. (d) PFM phase image on a 40 nm thick BTO flake after ferroelectric domain engineering by pooling a box-in-box pattern with tip voltages of +5 V and −5 V. (e) Local PFM amplitude (orange) and phase (black) hysteresis curves acquired on the transferred BTO flake.

Functional characterization of the as-isolated BTO layers was performed by Piezoresponse Force microscopy (PFM) to assess the preservation of the ferroelectric character and its switchable nature with applied electric fields. For this purpose, the BTO layers were transferred to Au-coated SiO_2_/Si substrates (bottom electrode) while the conductive AFM-probe is used as the mobile top electrode. Figure 2d shows a PFM phase map for a BTO sample of similar characteristics as those used for device fabrication subjected to domain writing by patterning a box-in-box design with opposite tip voltages (+5 and −5 V) above the coercive voltages. This result demonstrates that the ferroelectricity in the BTO films survives even in freestanding (peeled off from the parent epitaxy template substrate) form. Figure 2e shows a representative hysteresis loop on a selected location of the BTO flake. The reversal of the phase signal and the butterfly like amplitude loop are the footprint of the 180° polarization switching. The phase loop is quite symmetric with respect to the sign of the applied voltage. From similar cycles acquired at different locations of the same flake (see Supporting Information Figure S7) we obtain coercive voltages of 3.1 ± 0.6 V and −2.4 ± 0.3 V for the positive and negative regions, respectively. Asymmetric loops had also been measured in some of the flakes of different thickness (Supporting Information Figure S7), presenting a shift to the negative applied tip voltages. This negative imprint corresponds to a preferred down-polarization state of the BTO, which was also found for similar heterostructures using nonfreestanding BTO thin films.^51^

In the following, we show that the robust ferroelectric state of the single crystalline freestanding BTO layers can be used in combination with single-layer MoS_2_ layers to engineer a field effect device. For the device fabrication, a piece of Nitto SPV 224 tape is adhered onto the Gel-Film substrate with the thin BTO layer and peeled off suddenly. This leads to the transfer of smaller area (30 × 30 μm^2^, approx.) “flakes” of BTO that have more suitable dimension for our device geometry. We have explored two different approaches to deterministically transfer the BTO flakes that will be used as dielectric between two gold electrodes prepatterned on a SiO_2_ (285 nm)/Si substrate (Figures 3a and 3b): a deterministic transfer based on the use of nail polish polymer and one based on deterministic transfer directly from the Nitto-tape (see Materials and Methods). We note that although it is possible to create devices directly on BTO on an epitaxial growth substrate, our freestanding transfer on to SiO_2_ will allow development toward for more complex device architectures (dual-gated devices) and easier extraction of device properties, such as mobilities calculated below.

**Figure 3:**
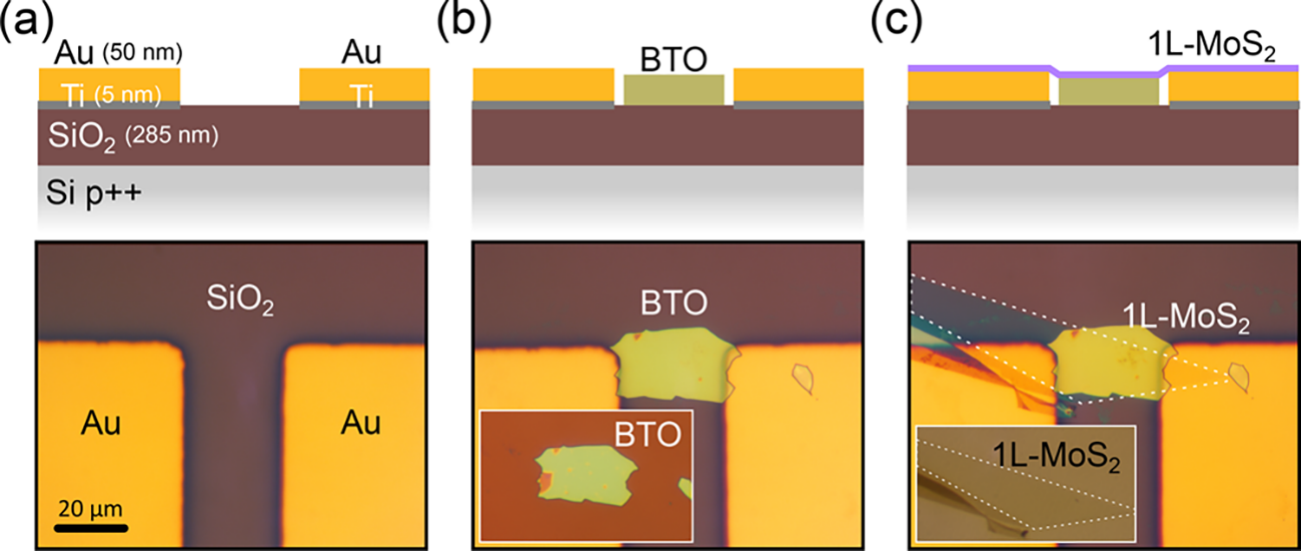
Fabrication of field-effect devices integrating single-layer MoS_2_ channels and a BTO dielectric. The bottom panels show optical microscopy images at different steps of the fabrication. The top panels show a cartoon with the cross section of the corresponding optical microscopy images to further clarify the geometry of the devices. (a) Two prepatterned gold electrodes onto a SiO_2_/Si substrate. (b) A 50 nm BTO flake is picked up from a carrier SiO_2_/Si substrate (inset in bottom panel) and transferred in between the gold pads. (c) A single-layer MoS_2_ flake is transferred from a Gel-Film carrier substrate (see inset in bottom panel) onto the BTO flake and bridging the two prepatterned gold electrodes.

The semiconductor channel is then fabricated by transferring a single-layer MoS_2_ flake onto the BTO flake and bridging the two prepatterned gold electrodes leading to a van der Waals mediated electrical contact (Figure 3c). Briefly, a bulk natural molybdenite mineral (Molly Hill Mine, Quebec, Canada)^52^ was cleaved with Nitto SPV 224 tape. Then the tape containing the cleaved micro crystals was put in contact to a Gel-Film surface and peeled off gently to leave some atomically thin flakes on the Gel-Film substrate. The surface of the Gel-Film is then inspected under an optical microscope (Motic BA 310 MET-T) in transmission mode to identify suitable single-layers of MoS_2_ (see inset in Figure 3c). Their thickness is first assessed by their apparent transmittance^53^ and then double-checked by microreflectance spectroscopy to verify single-layer flakes.^54,55^ Once a suitable single-layer MoS_2_ flake is identified it is transferred with the all-dry viscoelastic deterministic placement method.^56,57^ Note that, when the single layer MoS_2_ flake is connected to a multilayered portion, a laser trimming process was carried out to ensure that all the electrical transport occurs through the single-layer MoS_2_ (see Materials and Methods and Supporting Information Figures S8 and S9).^58^

The devices are electrically characterized under high-vacuum conditions (∼10^–6^ mbar) in a home-built vacuum probe station system.^59^ An in situ annealing at 200 °C for 2 h at high-vacuum (∼10^–4^ to 10^–5^ mbar) was performed prior to electrical transport measurements to improve the gold-MoS_2_ contact and to remove atmospheric adsorbates. Figure 4a shows source-drain current, at a fixed *V*_bias_ = 5 V, while the gate voltage is swept from −50 V to +50 V (at 1 V/s rate). See the Supporting Information Figures S10 and S11 for measurements on devices with hBN and BTO dielectrics at different gate sweeping rates (from 0.1 V/s to 10 V/s). The inset in Figure 4a shows a collection of *I*_ds_ vs *V*_bias_ curves (*IV*s hereafter) acquired at different gate voltages. From the slope of the *I*_sd_ vs *V*_g_ (*IV*_g_ hereafter) shown in Figure 3a one can extract the field-effect mobility of the transistor^60^
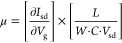
where ∂*I*_sd_/∂*V*_g_ is the slope of the *IV*_g_ trace, *L*, and *W* are the length and width of the semiconductor channel, respectively, *C* is the capacitance per unit area between channel and backgate electrode and *V*_sd_ is the source-drain bias. In our geometry, the capacitance can be calculated as two parallel plate capacitors in series, one with SiO_2_ and another one with BTO as dielectrics
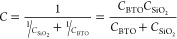
where *C*_*X*_ = ε_0_·ε_*x*_/*d*_x_ with ε_0_ the vacuum electrical permittivity and ε_*x*_ and *d*_*x*_ the relative permittivity and the thickness of medium “*x*” (SiO_2_ or BTO) respectively. Because of the very high ε_r_ expected for BTO (∼4000),^61−63^ in this geometry the capacitance will be strongly dominated by the SiO_2_ contribution. This is advantageous to extract accurate field-effect mobility values even in the case of not knowing the exact dielectric value of the BTO ultrathin layer. We have fabricated a parallel plate capacitor using a freestanding BTO layer as dielectric to experimentally determine its dielectric constant (see Supporting Information Figure S12) finding a value of ε_BTO_ = 4700 ± 500, in good agreement with the values reported for bulk BTO at room temperature.^61−63^ In Figure 4a we note that the additional structure in the *IV*_g_ curve (double hump feature) is a manifestation of the ferroelectric switching of the BTO layer. Similar features have been observed in devices with a ferroelectric layer.^35,37,64,65^

**Figure 4:**
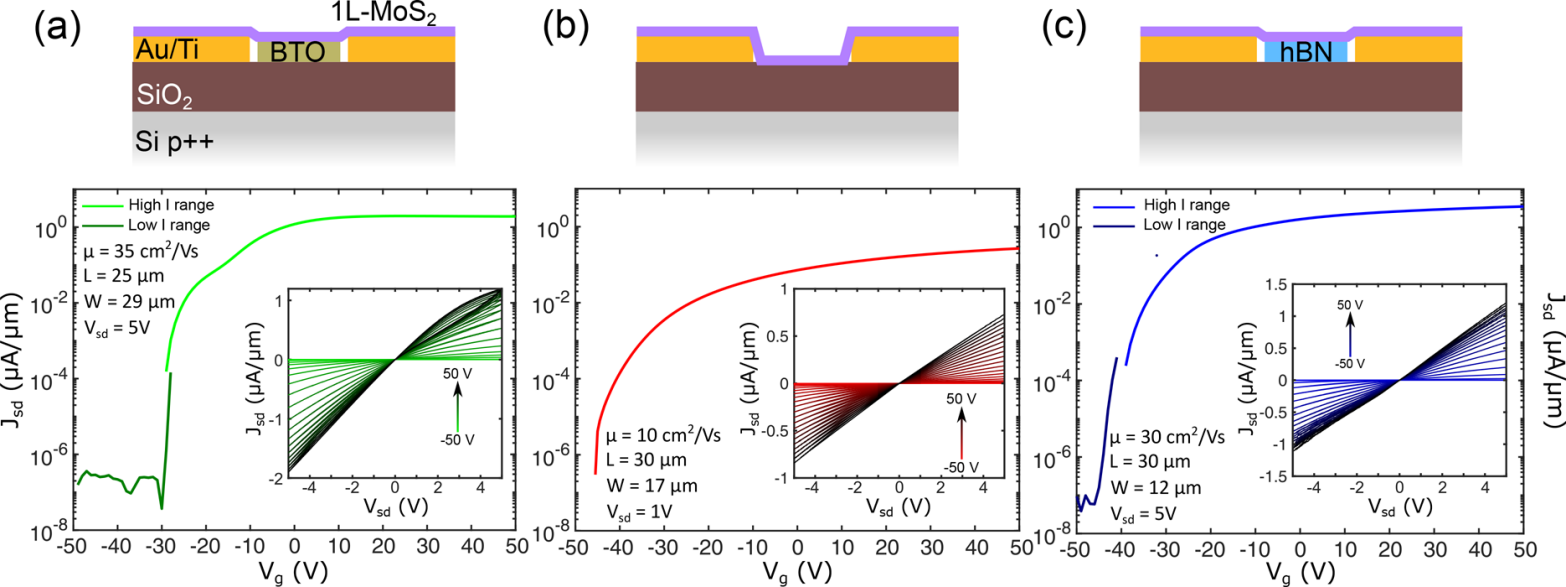
Gate tunable characteristics of 1L-MoS_2_ field-effect devices using BTO, SiO_2_ and hBN dielectrics. (a) Source drain current as a function of the gate voltage for a device integrating a BTO (48 nm)/SiO_2_ (285 nm) dielectric (see cartoon on top). (inset) Current vs drain-source bias voltage curves acquired at different gate voltages. (b) and (c) Similar data sets to (a) but collected for devices using a SiO_2_ (285 nm) dielectric layer and a hBN (∼30 nm)/SiO_2_ (285 nm) dielectric layer (see cartoons on top).

We fabricated and tested seven devices integrating a BTO dielectric finding two-terminal field-effect mobility values ranging from 5 to 70 cm^2^/(V s) (see the Supporting Information, Table S1 for a summary of the characteristics of all devices measured). Those values are substantially larger than those typically found on devices fabricated using the same protocol but with standard SiO_2_ dielectric (see Figure 4b) which are in the 0.1–10 cm^2^/(V s) range. Moreover, in order to put those large mobility values into context, we directly compare the performance of the MoS_2_ transistors integrating BTO dielectric with that of a device fabricated following the same steps but replacing the BTO flake by a hBN of similar thickness (Figure 4c). Note than hBN is used in 2D-based devices because it is considered as an almost ideal dielectric substrate due to its atomically flat surface and very low density of Coulomb scattering centers.^1,66^ Interestingly, the mobility of the BTO based devices is comparable to that of the hBN ones (1 to 80 cm^2^/(V s) for 4 measured devices) which we attribute to the very effective screening of the charged impurities due to the very high dielectric constant of BTO.^67,68^

Another important figure-of-merit of field-effect transistors is the current ON-OFF ratio, *I*_ON/OFF_, which provides information about how effectively the transistor can be switched OFF. Most of the BTO based devices show I_ON/OFF_ in the 10^5^ to 10^7^ range, comparable with the SiO_2_ (∼10^6^) or the hBN based transistors (∼10^5^ to 10^8^). The two BTO devices fabricated by nail polish transfer showed a poor switching performance (I_ON/OFF_ of ∼10) which we attribute to chemical doping of the MoS_2_ by the presence of traces of traces of nail polish that could not be removed with our current cleaning protocol. We are working on improving the cleaning process to avoid this issue, as nail polish transfer makes device assembly more straightforward than the deterministic Nitto tape based transfer, but that lays beyond the scope of the current work.

Given the ferroelectric character of BTO, it is important to study the transconductance curves both in forward and backward sweeps to draw our attention to the hysteresis. Figure 5a–c compares the current vs gate voltage curves acquired while sweeping the gate from −50 Vto +50 V and back to −50 V for MoS_2_ field-effect transistors with BTO, SiO_2_ and hBN dielectrics. The hysteresis of the BTO based devices ranges from 16 to 44 V, substantially larger than that of devices with nonferroelectric dielectrics where the hysteresis typically arises from the polarizability of adsorbed or trapped molecules on the surface or interfaces. Given the large difference in dielectric constant between the BTO layer (∼4700) and the SiO_2_ (∼3.9) one can expect that most of the gate voltage difference will occur across the SiO_2_ dielectric and thus the BTO film will not be in a fully saturated ferroelectric poled state. This, in combination with the presence of adsorbate traps at the different interfaces (see below) and the intrinsic in-plane polarization of the freestanding BTO flakes (see Figure S1), will lead to a depolarization of the devices over time. We address the reader to the Supporting Information Figure S13 for a data set of retention time measurements performed at different temperatures.

**Figure 5:**
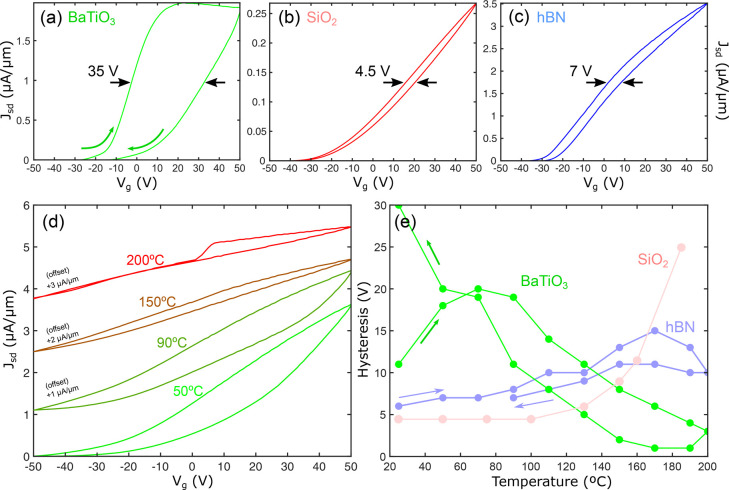
Characterization of the ferroelectric nature of the BTO dielectric. (a) to (c) Comparison between the hysteresis measured in the current vs gate voltage curves for BTO (48 nm)/SiO_2_ (285 nm) dielectric (a), SiO_2_ (285 nm) dielectric (b) and hBN (∼30 nm)/SiO_2_ (285 nm) dielectric (c). (d) Dependence of the current vs gate voltage curves as a function of the temperature. (e) Hysteresis of the current vs gate voltage curves as a function of the temperature for BTO, hBN, and SiO_2_ based devices.

While ferroelectric-induced modulation in transistors is expected to induce counterclockwise hysteresis loops,^69,70^ a clockwise hysteresis is observed in Figure 5a. This inversion is actually a relatively common result already reported in 2D FETs with ferroelectric dielectrics, first with a graphene channel^36,40,71^ but also in transition metal dichalcogenide based transistors (including MoS_2_).^39,65,70,72^ Most studies agree that there is a subtle interplay between the ferroelectric polarization and interfacial phenomena, attributing the unexpected clockwise hysteresis to the interfacial charge screening of ferroelectric polarization. Ultrathin oxide ferroelectrics like BTO are typically oxygen deficient, and the oxygen vacancies accumulate at the surface.^73^ The coupling of these oxygen vacancies to the ferroelectric polarization to compensate polarization charges could be responsible for polarization-assisted charge trapping at the BTO/MoS_2_ interface.^71,72^ Moreover, although our devices have been annealed in high-vacuum (200 °C for 2 h.) prior to electrical characterization, remaining adsorbates trapped at the interface between the semiconducting 2D material and the dielectric, that could potentially contribute to screening the ferroelectric polarization, would explain the clockwise hysteresis of the transfer curves.

To get further insight on the role of the BTO ferroelectric character on the electrical behavior of the devices, we performed temperature dependent analysis of the current vs gate voltage curves, to follow the evolution of the hysteresis. These measurements can help to identify the origin of the hysteresis as shown for SiO_2_-gated devices.^74^ In our BTO-based heterostructures, increasing temperature brings an additional interesting phenomena: the ferroelectric to paraelectric transition of the BTO (above 120 °C for bulk crystals).^75,76^ If the large hysteresis measured in BTO-based devices is related with the polarization of BTO, a change is expected when measuring above the transition temperature. Figures 5d and 5e present the transfer curves and the hysteresis (calculated as the difference between voltages at I_ON_/2 for the forward and backward sweeps in the current vs gate voltage curves, see arrows in Figure 5a to 5c, as a function of temperature. From 70 °C there is a decreasing trend of the hysteresis that almost vanishes above 150 °C, in good agreement with the expected ferroelectric to paraelectric transition. The larger hysteresis values are recovered upon decreasing the temperature back to room temperature. This behavior clearly differs from that observed in the hBN and SiO_2_-gated devices, which show an overall increase with temperature, frequently related to thermally activated oxide/adsorbate traps (close to the interface) that can capture and release electrons from MoS_2_.^74^ We address the readers to the Supporting Information Figure S14 for the complete data set of the hBN and SiO_2_ based devices and Figure S15 for another data set of BTO based devices.

In summary, we present the integration of thin BTO layers, delaminated from the substrates where they were epitaxially grown, as ferroelectric dielectrics in single-layer MoS_2_ field effect devices. Their high dielectric constant helps to screen out Coulomb scatterers leading to mobility values comparable to those obtained in devices integrating hBN dielectric, considered as an almost ideal substrate for high performance 2D-based devices in the nanomaterials community. In the devices integrating BTO layers we observe a robust hysteresis of the current vs gate voltage traces, very distinct with respect to the hysteresis observed when other dielectrics are used, which we attribute to the ferroelectric polarization switching of the BTO layer. A proof that the poling of the BTO layer is playing an important role in the hysteretic behavior is obtained from electrical characteristics at different temperatures, finding that the hysteresis nearly disappears at temperatures near the ferroelectric-to-paraelectric transition. Our results are the first step toward the fabrication of 2D-based devices with BTO based ferroelectric dielectrics and, in a more general perspective, toward the integration of a large family of transition metal oxides displaying strongly correlated physical phenomena in 2D-based devices.

## Materials and Methods

### Epitaxial Growth of BTO Thin Films

Fifteen nanometers of LSMO and 15–50 nm of BTO thick films were grown epitaxially onto (001) STO substrates by pure oxygen (3.2 mbar) sputtering technique at high temperature (900 °C). Both layers were grown sequentially without breaking vacuum. Interfaces were atomically sharp with LaO termination planes of the LSMO Manganite facing TiO_2_ planes of the BTO as shown by scanning transmission electron microscopy (STEM), combined with electron energy-loss spectroscopy (EELS). This growth mode yielded out of plane ferroelectric polarization, preferentially pointing downward.^73^

### Release of BTO Thin Films from Their Parent Substrate

The strained LSMO/BTO heterostructure is adhered to a commercial polydimethylsiloxane film (Gel-Film WF 4 × 6.0 mil by Gel-Pack) and immersed at room temperature in a diluted solution of 0.5 mL KI (3 mol/L) + 0.5 mL HCl (37%) and 10 mL deionized H_2_O, which dissolves the LSMO in an average time of 3 days and allows the delamination of the BTO layer without damaging its properties.

### Nail-Polish Deterministic Transfer of BTO

The Nitto tape with the BTO flakes is adhered to a SiO_2_/Si substrate and gently removed to transfer the BTO. The SiO_2_/Si acts as a carrier substrate followed by surface inspection by optical microscopy to find a suitable BTO flake. The flake is then picked-up with a nail-polish deterministic transfer method^77^ and placed between two gold electrodes prepatterned on a SiO_2_ (285 nm)/Si substrate.

### Deterministic Transfer from Nitto Tape of BTO

we select the BTO flake to be transferred by optical microscopy inspection of the Nitto tape surface. The Nitto tape is then mounted into a deterministic placement setup to align the selected BTO flake between two gold electrodes prepatterned on a SiO_2_ (285 nm)/Si substrate. By gently pressing the tape against the substrate and peeling off very slowly with the help of a micrometer manual actuator of the transfer setup one can transfer the BTO flake to the desired location.

### Scanning Transmission Electron Microscopy

STEM-EELS characterization was carried out using a JEOL JEM-ARM 200cF aberration corrected electron microscope operated at 200 kV, equipped with a cold field emission gun and a Gatan Quantum spectrometer. BTO freestanding flakes were transferred onto a holey Si_3_N_4_ membrane for STEM-EELS observation using the deterministic transfer method described above.

### Piezoresponse Force Microscopy Measurements

PFM measurements were carried out using a commercial AFM system and software from Nanotec^78^ operating in ambient conditions. SiO_2_/Si substrates with an e-beam evaporated film of 5 nm Ti and 15 nm Au were used for the PFM measurements. PtIr-coated commercial tips from Nanosensors (PPP-NClPt) were used and *V*_DC_ and *V*_AC_ voltages were applied to the tip while the sample was grounded. The drive frequency, drive amplitude (*V*_AC_), and trigger force were ∼52 kHz, 1–3 V, and 200–500 nN, respectively. Hysteresis loops were measured in the spectroscopy mode at the selected locations. A sequence of DC voltages was applied to the tip (*V*_DC_), with the *V*_AC_ voltage superimposed to excite the electromechanical vibration of the sample. Both, phase and amplitude of the vibration are recorded as a function of *V*_DC_ voltage. Amplitude signal yields information on the magnitude of the electromechanical vibration whereas phase signal relates to the orientation of the out-of-plane component of the polarization (upward or downward). Ferroelectric domain engineering was performed by poling box-in-box patterns with *V*_DC_ tip voltages above the coercive voltages and the *V*_AC_ modulation off. Subsequent PFM imaging shows the phase contrast due to 180° switching of the polarization, and the amplitude signal, proportional to the magnitude of surface displacement induced by the converse piezoelectric, showing a minimum between both domains.

### Electrode Deposition

Prepatterned gold electrodes were fabricated by evaporation of 5 nm Ti (used as adhesion layer) and 45 nm Au onto a SiO_2_ (285 nm)/Si (p++) substrate through a shadow mask (Ossila, E321) in an electron-beam evaporator system.

### Laser Trimming of the MoS_2_ Flakes

Laser-cutting of MoS_2_ flakes were carried out in ambient environment with a confocal Raman microscopy system (MonoVista CRS+ from Spectroscopy & Imaging GmbH) using a 532 nm excitation laser with an incident power of 28.6 mW and a 100× objective with an integration time of 10 s.^58^

### Electrical Characterizations

All the electrical characterization of devices were carried out in a home-built high-vacuum (∼10^–6^ mbar, *T* = 20–200 °C) chamber. A source-meter unit (Keithley 2450) was used for performing the electrical measurements between source and drain electrodes. Two TENMA programmable benchtop power supplies (ref 72–2715) are connected in parallel to perform gate voltage sweeps between −50 V and +50 V.

## Supporting Information Available

Lattice constants determination from high resolution STEM images; thickness determination of thin BTO flakes using apparent optical color; optical method to determine the thickness of BTO flakes through quantitative analysis of their optical contrast; Raman and photoluminescence spectroscopy of MoS_2_ on BTO; X-ray diffraction to determine the out-of-plane lattice constant and estimate the thickness of freestanding BTO layers; comparison between the thickness determined by different techniques for the LSMO/BTO stack and the BTO film before and after releasing as a function of the growing time; local PFM amplitude and phase hysteresis curves acquired on additional transferred BTO flakes; process of laser trimming to ensure that the electrical transport occurs along the 1L-MoS_2_ and the multilayer part is electrically isolated from the leads; scanning photocurrent microscopy measurement performed to demonstrate that the laser-cut process effectively disconnected the 1L-MoS_2_ part from the multilayer flake; current vs gate voltage sweeps performed at different gate sweeping speeds for hBN and BTO based FET devices; experimental determination of the dielectric constant of freestanding BTO films. Temperature dependence of the retention time; temperature-dependent transfer curves and hysteresis of nonferroelectric based MoS_2_ FETs; temperature-dependent transfer curves and hysteresis of another BTO based MoS_2_ FET (BTO_14 device); summary of device characteristics for all devices measured (PDF)
