# Supplementary material for: Combining Freestanding Ferroelectric Perovskite Oxides with Two-Dimensional Semiconductors for High Performance Transistors
Source: Nano Lett. 2022 Sep 15;22(18):7457–66. doi: 10.1021/acs.nanolett.2c02395 (PMC9523702; doi:10.1021/acs.nanolett.2c02395)
Supplement: Supplementary file 1 — nl2c02395_si_001.pdf [file nl2c02395_si_001.pdf]

Supporting Information:

Combining freestanding ferroelectric perovskite oxides with two-dimensional semiconductors for high performance transistors

Sergio Puebla[1], Thomas Pucher[1], Victor Rouco[2], Gabriel Sachez-Santolino[2,3,4], Yong Xie[1,5], Victor Zamora[2], Fabian A. Cuellar[2], Federico J. Mompean[1,3], Carlos Leon[2,3], Joshua O. Island[6], Mar Garcia-Hernandez[1,3], Jacobo Santamaria[2,3], Carmen Munuera[1,3*], Andres Castellanos-Gomez[1,3*]*

[1]Materials Science Factory. Instituto de Ciencia de Materiales de Madrid (ICMM-CSIC), Madrid, E-28049, Spain.

[2] GFMC. Dept. Fisica de Materiales. Facultad de Fisica. Universidad Complutense. 28040 Madrid, Spain.

[3] Unidad Asociada UCM/CSIC, "Laboratorio de Heteroestructuras con aplicación en spintrónica"

[4] Instituto Pluridisciplinar, Universidad Complutense de Madrid, 28040 Madrid, Spain.

[5] School of Advanced Materials and Nanotechnology, Xidian University, Xi'an, 710071, China

[6] Department of Physics and Astronomy, University of Nevada Las Vegas, Las Vegas, NV, 89154, USA

jacsan@ucm.es , cmunuera@icmm.csic.es , andres.castellanos@csic.es

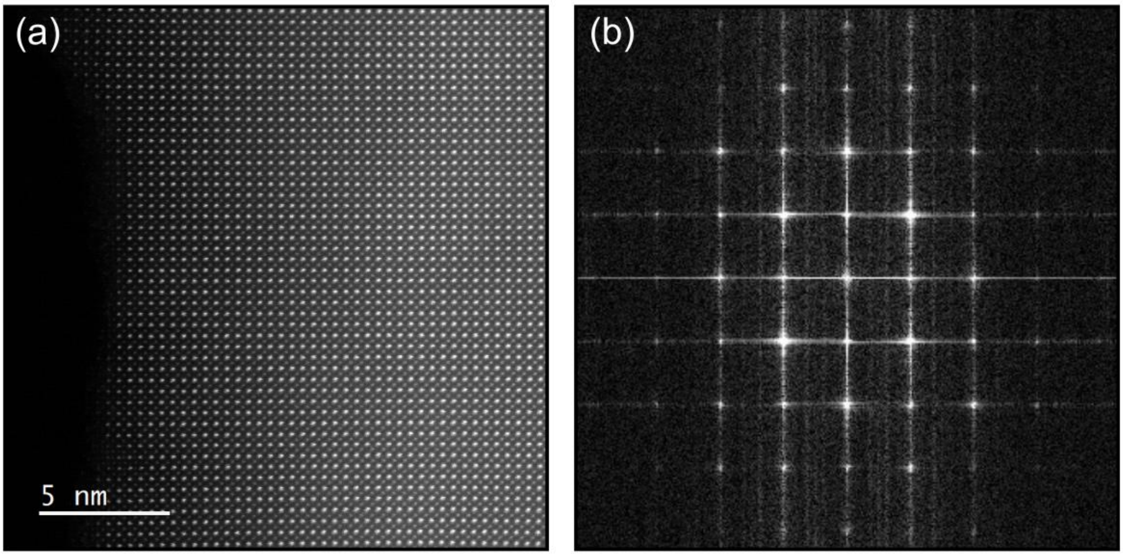

Figure S1. Lattice constants determination from the FFT (right panel) from the high resolution STEM image on the left. We obtain 4.10 and 4.13 Å for the in-plane lattice parameters along the high symmetry (x and y) directions of the tetragonal ferroelectric structure. These values are somewhat larger than the expected bulk in plane values (4 Å). This probably results from the in-plane polarization in the freestanding layers.

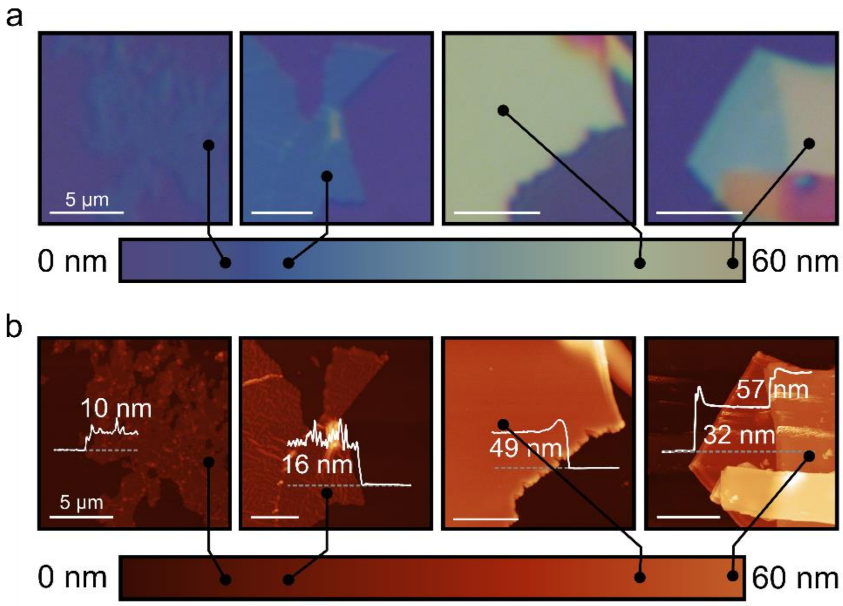

Figure S2. Thickness determination of thin BTO flakes using apparent optical color. (a) Optical microscopy images of BTO flakes on 285 nm of SiO2 on Si substrates. The change in color corresponds to a change in the flake thickness. (b) AFM scans of the same flakes as in panel (a).

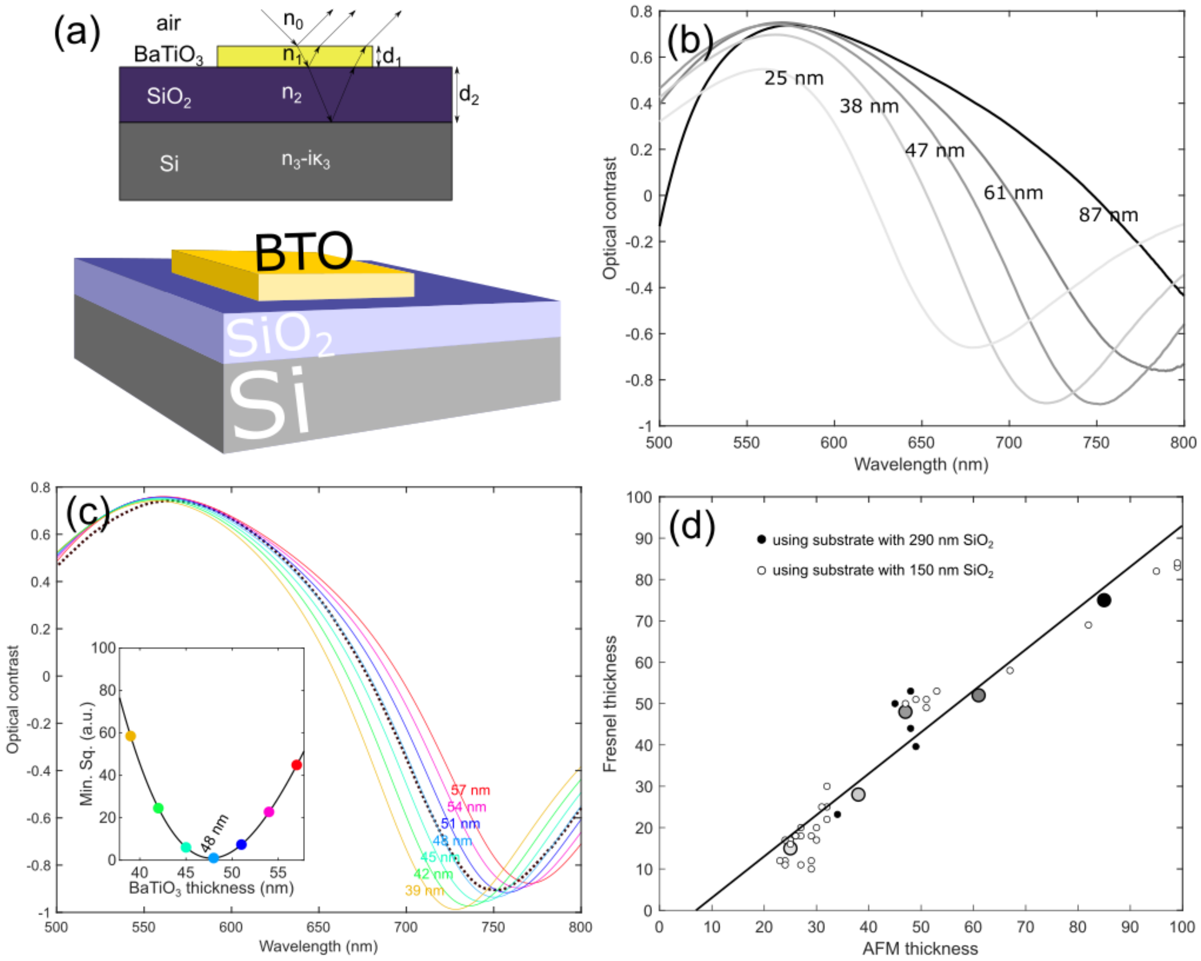

Figure S3. Optical method to determine the thickness of BTO flakes through quantitative analysis of their optical contrast. (a) Optical model used to calculate the BTO optical contrast. (b) Acquired optical contrast for 5 different BTO films with thickness ranging from 25 nm to 87 nm (as determined by AFM). The optical contrast spectra have a dip feature that depends monotonically with the thickness of the BTO film. (c) Comparison between an experimental optical contrast spectrum (dotted line) and the results of optical contrast calculations assuming different thicknesses of the BTO film. A best-fit is obtained assuming a BTO film thickness of 48 nm. The refractive index used for BTO in the optical contrast calculation is obtained from Ref. [S1]. (d) Comparison between the thickness determined through the optical model based on the Fresnel law and on the AFM measurement. The agreement between the two thicknesses is rather good (the straight line is a guide to the eye with slope 1) although there is a systematic error that could be due to artifacts in the AFM measurements due to the presence of adsorbates or to a slight difference in the refractive index of freestanding BTO with respect to the reported bulk value in the literature.

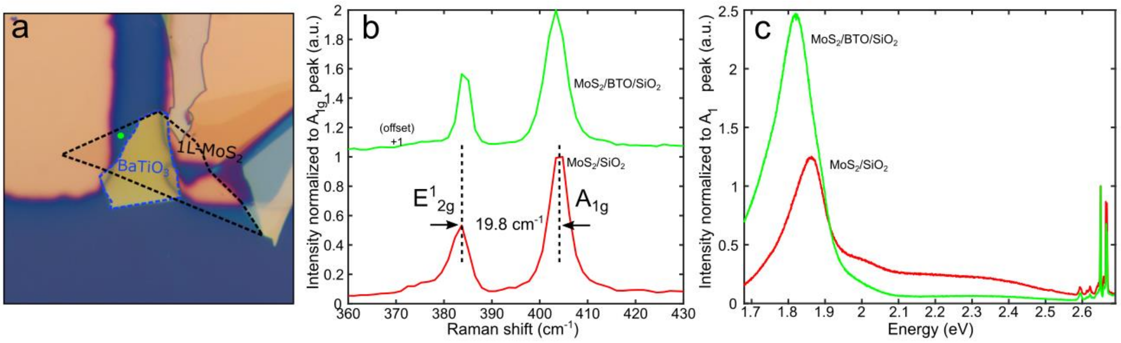

Figure S4. Raman and photoluminescence spectroscopy of MoS_2 on BTO. (a) Optical image of one of the devices with MoS_2 on BTO. The green dot shows the location of the laser for the measurement of MoS_2 on SiO_2. (b) Raman spectrum of MoS_2 on SiO_2/Si and BTO/SiO_2/Si substrates. (c) Photoluminescence spectra of MoS_2 on BTO/SiO_2/Si and on SiO_2/Si. The photoluminescence spectra on top of BTO shows a more prominent contribution from the neutral A excitons than the spectra onto SiO_2 and an enhanced photoluminescence. Note that the intensity has been normalized to the A_{1g} Raman peak to account for the difference in optical absorption in the two measurements. Note that the photoluminescence of single-layer MoS_2 flakes is usually dominated by one broad peak around 1.8-1.9 eV attributed to the generation of the neutral A excitons and charged trions (A- exciton) [S2]. The A- exciton occurs at lower energy than the neutral A exciton. Therefore, the MoS_2/BTO sample displays a photoluminescence at lower energies because of a higher contribution of the trion generation. This indicates that the polarization of the underlying BTO effectively electrostatically dopes the MoS_2 facilitating the generation of trions (in good agreement with the field-effect characterization measurements).

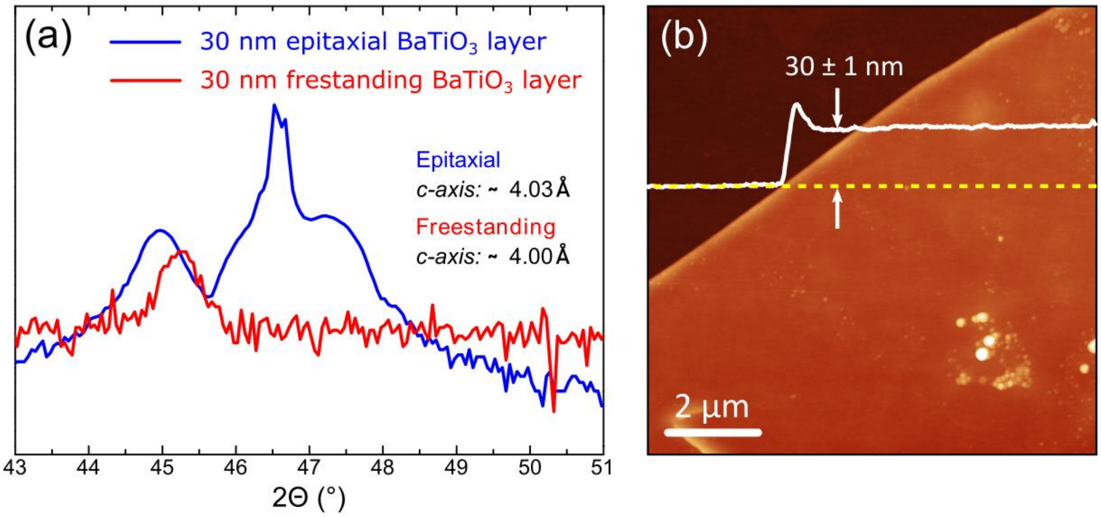

Figure S5. (a) X-ray diffraction pattern of an epitaxial BTO film on a LSMO buffer layer deposited on (001) oriented STO (blue line). The brown line shows the same spectrum measured on the (same) freestanding BTO layer. Notice the shift of the (002) peak to higher angles indicating the reduction of the out-of-plane lattice constant. The BTO layer thickness (d = 28 ± 3 nm) was also estimated from the half width at maximum height FWMH of the (002) diffraction peak (b) for both the epitaxial and the freestanding layer using Scherrer's formula ($d = 0.9\ \lambda_x/b \cos(\theta)$). λ_x stands for the X-ray wavelength and θ for the angle of the diffraction peak. (b) AFM topography of the same BTO film after delamination and transfer to a SiO_2/Si substrate.

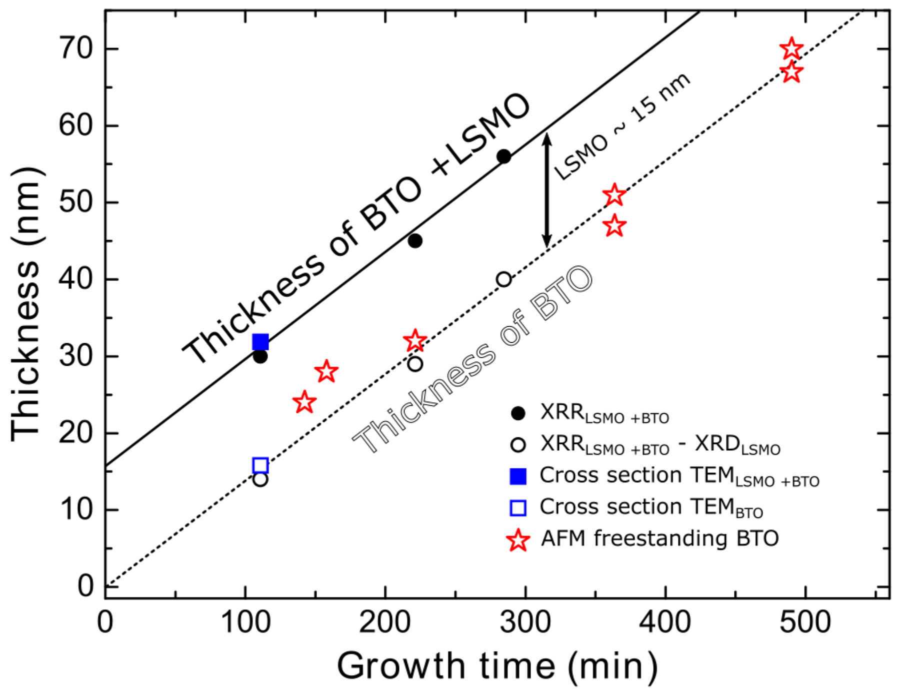

Figure S6. Comparison between the thickness determined by different techniques for the LSMO/BTO stack (filled markers) and the BTO film (empty markers) before and after releasing as a function of the growing time. The thickness of the BTO/LSMO stack is determined by X-ray reflectivity (XRR), see filled circles. The thickness of the BTO film prior-release can be determined by subtracting the thickness of the LSMO film (determined separately by X-ray diffraction, XRD) to the thickness of the stack (see empty circles). The empty stars show the AFM thickness of BTO films once released from the LSMO and transferred onto a SiO_2/Si substrate. Cross sectional TEM has been also used in one sample to directly measure the thickness of the LSMO/BTO stack and the LSMO and BTO layers.

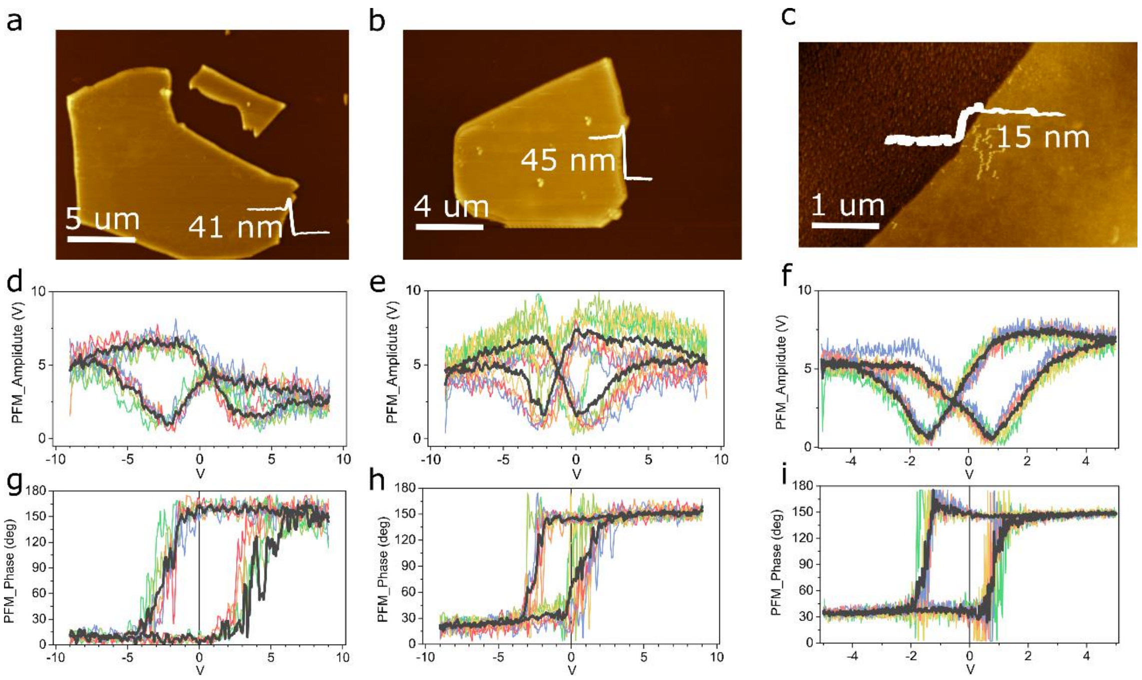

Figure S7. Local PFM amplitude and phase hysteresis curves acquired on additional transferred BTO flakes. (a-c) AFM topography scans of the flakes. (d-f) Local PFM amplitude for the same flakes shown in panels (a-c). (g-i) Local PFM phase for the corresponding flakes in (a-c). The darker black curves in (d) to (i) are the average over the individual hysteresis curves acquired on the flakes.

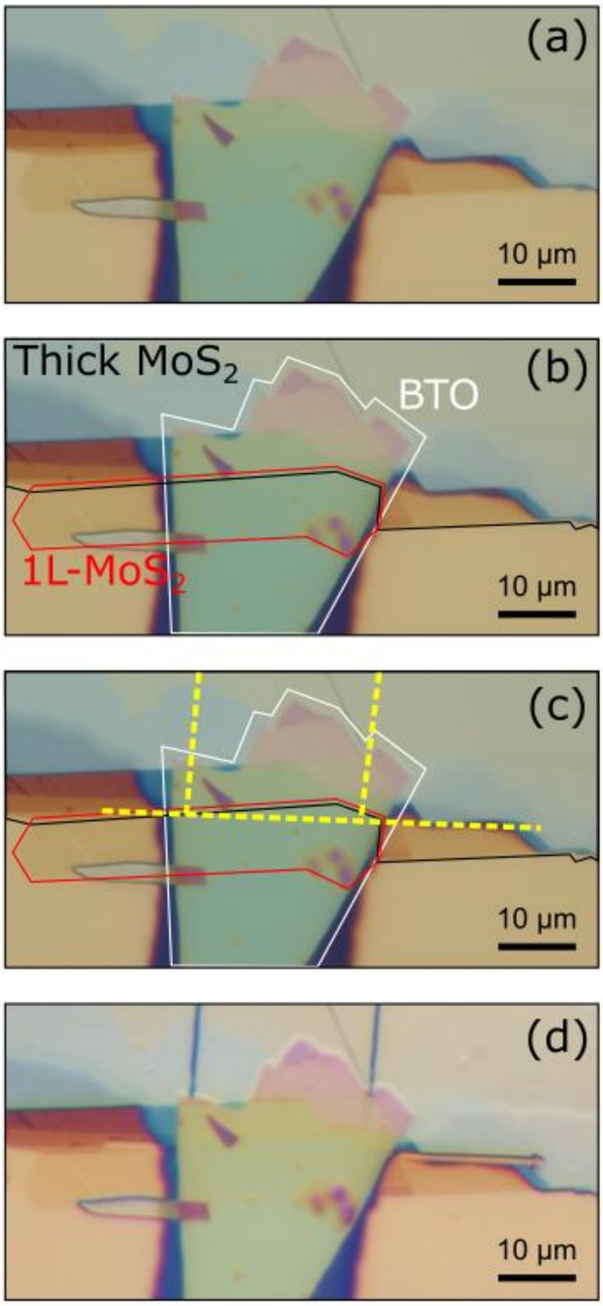

Figure S8. Pictures illustrating the process of laser trimming to ensure that the electrical transport occurs along the $1L\text{-}MoS_2$ and the multilayer part is electrically isolated from the leads. (a) and (b) example of one of the $1L\text{-}MoS_2$ devices that was initially connected to a multilayer part. (c) Scheme of the laser-cuts used to isolate the multilayer part. (d) Optical microscopy image of the flake after the laser cut process. The cuts are easily visible in the multilayer parts of the flake.

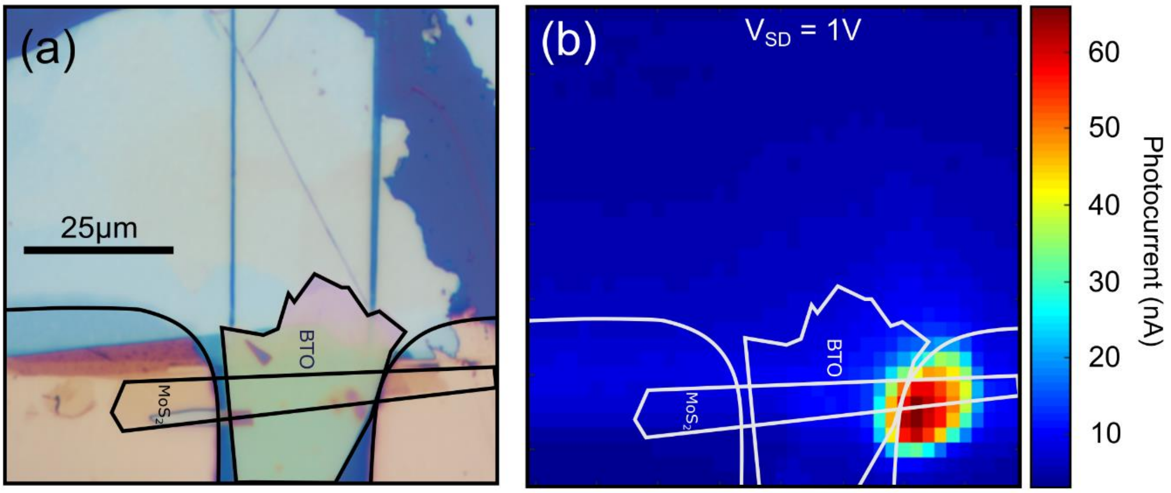

Figure S9. Scanning photocurrent microscopy measurement performed to demonstrate that the laser-cut process effectively disconnected the 1L-MoS_2 part from the multilayer flake. (a) Optical microscopy image of the flake after the laser cut process. The cuts are easily visible in the multilayer parts of the flake. (b) Scanning photocurrent map where one can observe that the multilayer part of the flake, despite of being more light-absorbing does not contribute to the photocurrent generation. All the current is being generated at the interface between the 1L-MoS_2 flake and the metal electrode as expected from a device with a certain Schottky barrier.

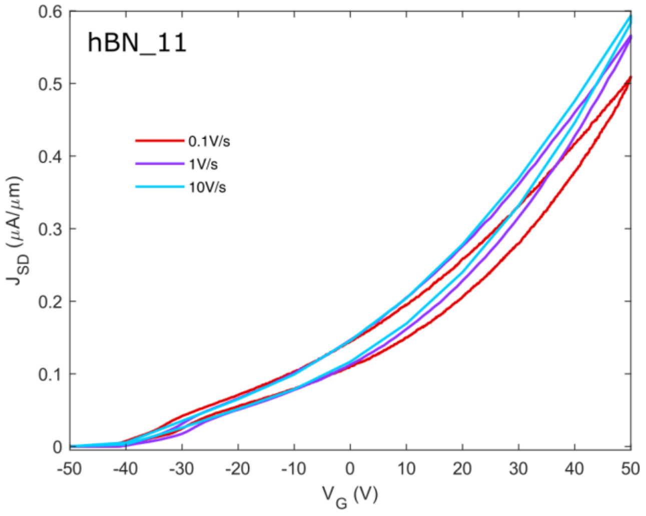

Figure S10. Current vs. gate voltage sweeps performed at different gate sweeping speeds, from 0.1 V/s to 10 V/s on the sample with hBN dielectric hBN_11.

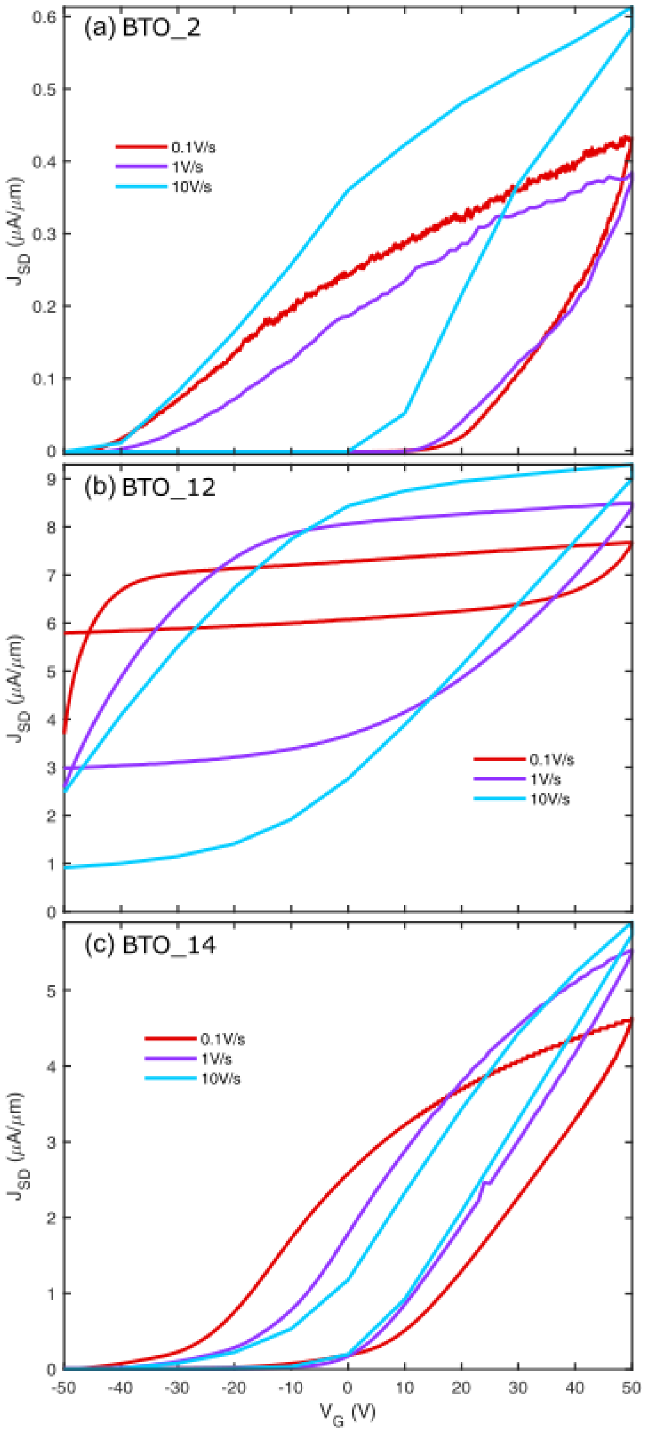

Figure S11. Current vs. gate voltage sweeps performed at different gate sweeping speeds, from 0.1 V/s to 10 V/s. Samples in (a) and (c) are prepared with Nitto tape method, and sample in (b) is prepared with Nail polish method and it presents a lower ON/OFF ratio, probably due to the presence of adsorbate contaminants in the BTO/MoS_2 interface because of an incomplete removal of the nailpolish with our current cleaning recipe (see discussion in the Main Text).

Experimental determination of the dielectric constant of freestanding BTO films:

By fabricating a parallel plate capacitor structure out of Au top and bottom contacts and a BTO flake in between we are able to calculate the dielectric constant by measuring the capacitance of the flake with a capacitance meter. The bottom contact is fabricated by employing photolithography and subsequent evaporation of Au. The BTO flake is then transferred on top of the bottom contact via the previously used nail polish method. Finally, a strip of gold, previously fabricated on a Si/SiO_2 substrate (applying the same

lithography/evaporation procedure), is picked up with a Gel-Film stamp (a commercial polydimethylsiloxane film) and transferred on top of the BTO flake bridging it to a contact pad.

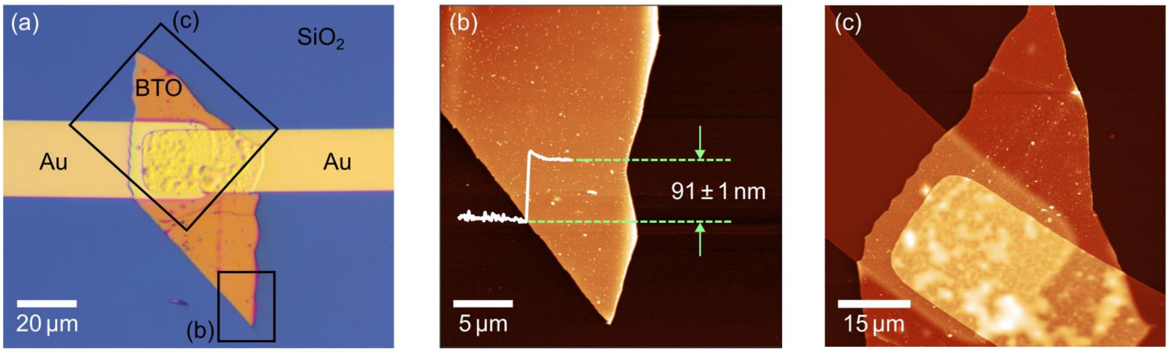

Figure S12. Experimental determination of the dielectric constant of freestanding BTO. (a) Microscope image of the finished capacitor structure showing both Au contacts and the BTO flake in between, enclosing an overlapping area of 23×25 μm^2. The thickness of the BTO flake is measured by AFM, with the result depicted in (b), presenting a thickness of 91 nm. Image (a) in combination with an AFM image of the junction (c) justify the use of the parallel plate capacitor model as the chosen fabrication method results in a clean capacitor structure with well-defined borders and therefore minimal stray field capability.

Using the formula for a parallel plate capacitor the dielectric constant of the BTO flake can be calculated as

$$\varepsilon_r = \frac{C \cdot t}{\varepsilon_0 \cdot A},$$

with C the measured capacitance, t the thickness of the flake, ε_0 the vacuum permittivity ($8.85 \cdot 10^{-12}$ F/m) and A the overlapping area.

To measure the capacitance of the flake, the structure is first connected to a 22 kΩ resistor for 10 s to get rid of any remaining charge. Subsequently the two gold pads are connected to a capacitance meter, reading out the capacitance value.

In our case the overlapping area is defined as 23×25 μm^2 and the AFM measurement provides a thickness determination of 91 ± 1 nm. By connecting the two gold contacts to a capacitance meter a capacitance of 264 ± 1 pF is measured, resulting in a dielectric constant $\varepsilon_r = 4700 \pm 500$, in good agreement with the dielectric constant value measured for bulk BTO at room temperature (~4000).

Retention studies were carried out for a BTO-based device as a function of temperature. The retention properties were evaluated after polarizing the BTO in the up (↑) or down (↓) state by gating either at *V*g =+50V or *V*g =-50V. After the corresponding gate pulse, the source-drain current at *V*bias = 1V and *V*g =0V was measured as a function of time. All measurements were performed in high vacuum (10^{-6} mbar) and in dark conditions. Figure S13(a) shows the evolution for both states at room temperature. The ratio of current in the on-state (P↓) to off-state (P↑) is initially ≈10 and current relaxation is observed immediately after pulsed-gating for both states. As in the case of the hysteresis cycle inversion, the interplay between ferroelectric polarization and interfacial phenomena also affect the evolution of the retention curves. Similar relaxation behavior has been reported in 2D-based devices with ferroelectric films as dielectrics and ascribed to the dissipation of interfacial charges over time.[S3,S4] Moreover, the freestanding BTO films present an intrinsic in-plane polarization (see the discussion related to Figure S1). Although the system can be poled in the out-of-plane direction (see the PFM analysis in Figure 2 of the main text and Figure S7 of the Supporting Information) it is expected that the system will evolve towards an in-plane polarization state. And finally, given the large difference in dielectric constant between the BTO (~4700) and SiO_2 (~3.9), one might expect that most of the voltage difference will occur across the SiO_2 dielectric and thus the BTO film will not be in a fully saturated ferroelectric switched state. All these mechanisms can contribute to the de-polarization of the device over time.

To estimate the retention time, the experimental data in Figure S13(a) was fit to a bi-exponential curve and extrapolated to determine the crossing point between the two states. The retention characteristics were further analyzed at temperatures close to (Fig. S13(b)) and above (Fig. S13(c)) the BTO ferroelectric to paraelectric transition. In the vicinity of the transition, the initial current ratio between up and down polarized states is considerably reduced, to ≈1.7, as is the time elapsed to the crossing point. At 150°C, BTO polarization is not expected to play a role in the device characteristics, which has a clear impact in the retention properties. Figure S13(c) shows a negligible difference between the current measured after gating at *V*g =+50V and *V*g =-50V. Figure S13(d) presents the extrapolated values of the crossing points as a function of temperature. Following a trend similar to the hysteresis of the transfer curve, there is a decreasing tendency with temperature and retention vanishes above 120°C concomitant with the paraelectric transition of the BTO flake.

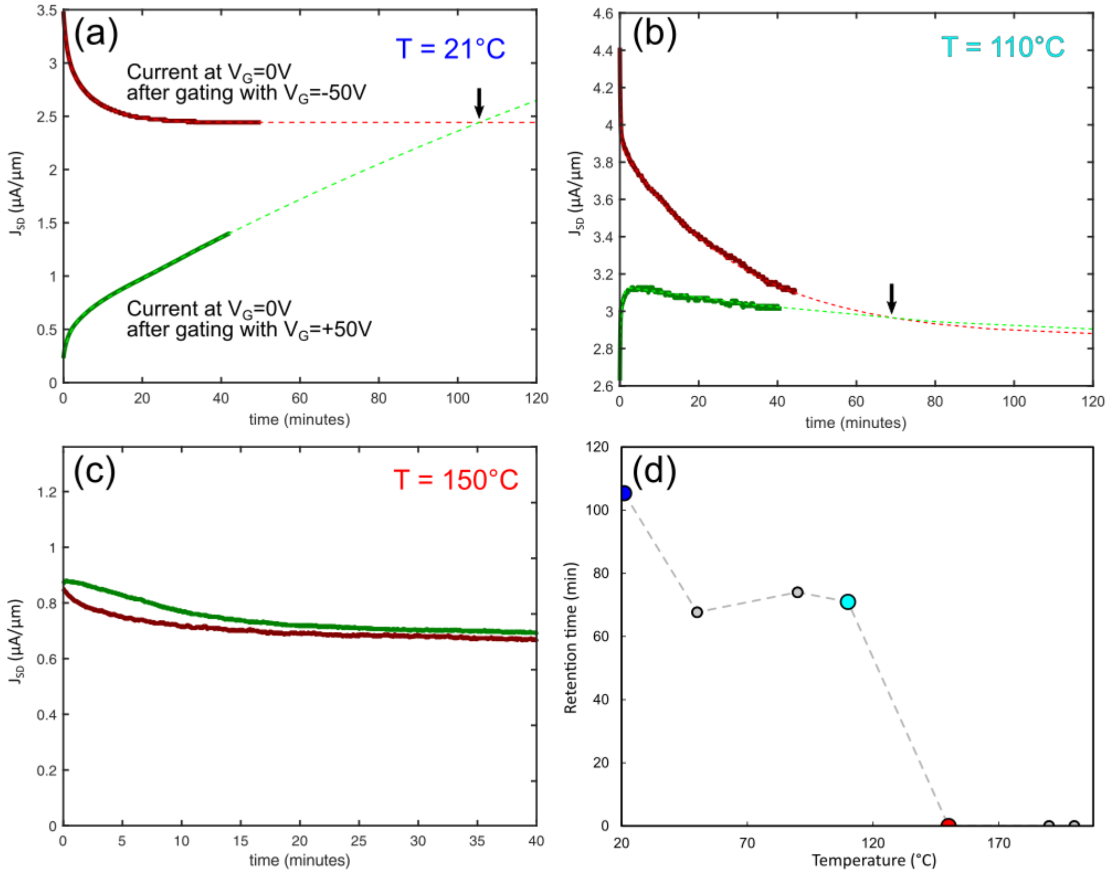

Figure S13. (a)-(c) Retention characteristics as a function of temperature for 1L-MoS_2 field-effect device (BTO_14) using BTO (~50 nm)/SiO_2 as dielectric. Experimental data was fit to a bi-exponential curve and extrapolated (dashed lines) to estimate the retention time from the crossing point between the two state curves, marked with arrow. (d) Evolution with temperature of the retention time, defined as the crossing point of both states.

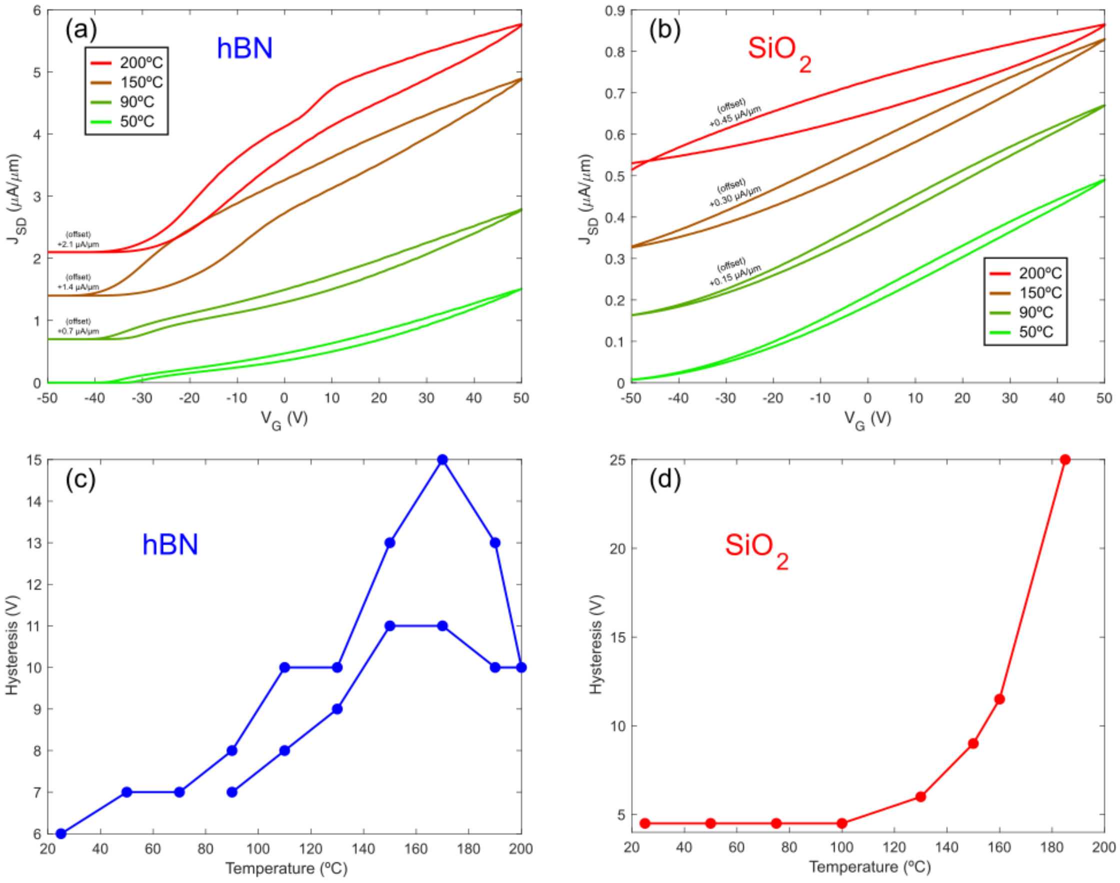

Figure S14. Temperature dependent transfer curves and hysteresis of non-ferroelectric based MoS_2 FETs. (a) and (b) Dependence of the current *vs*. gate voltage curves as a function of the temperature for single-layer MoS_2 devices with hBN and SiO_2 dielectrics. (c) and (d) Hysteresis of the current vs. gate voltage curves as a function of the temperature.

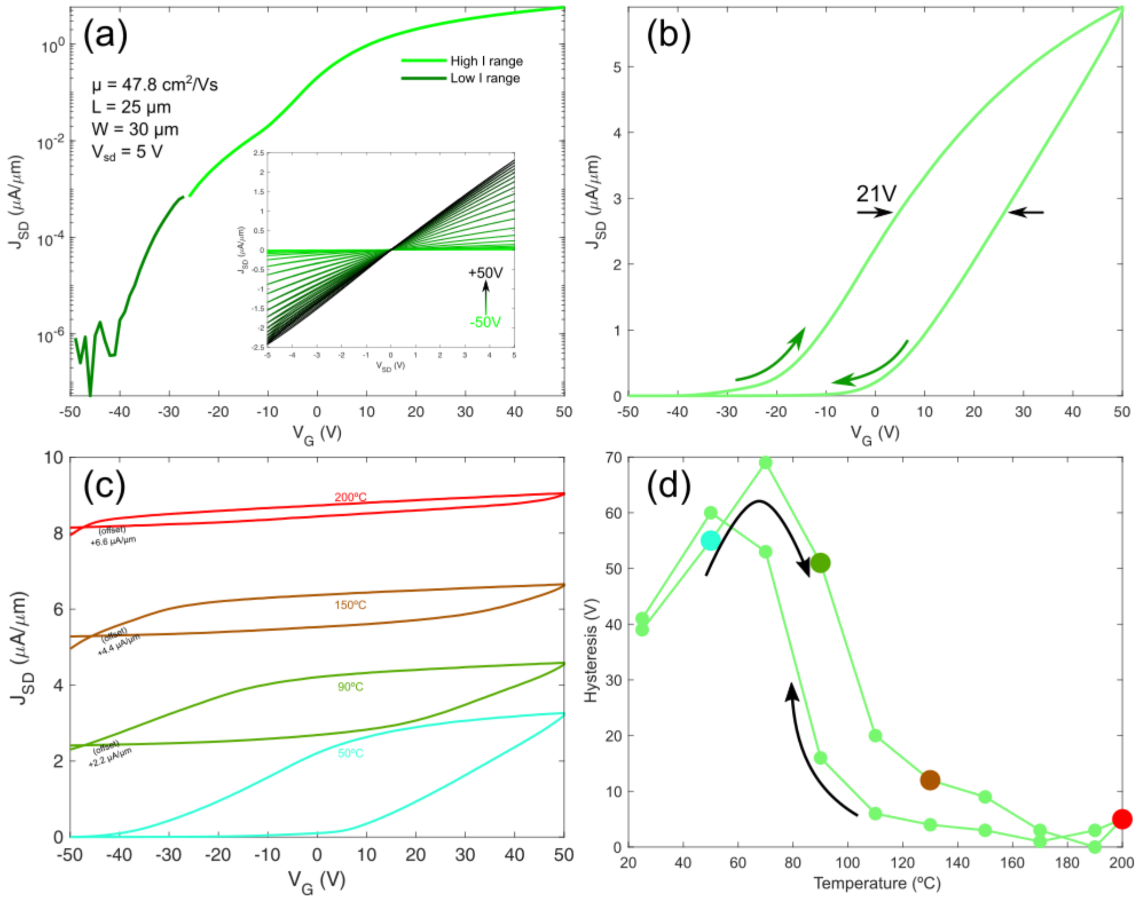

Figure S15. Temperature dependent transfer curves and hysteresis of another BTO based MoS_2 FET (BTO_14 device). (a) Source drain current as a function of the gate voltage for a device integrating a BTO (55 nm)/SiO_2 (285 nm) dielectric (see cartoon on top). (inset) Current vs. drain-source bias voltage curves acquired at different gate voltages. (b) Hysteresis measured in the current *vs*. gate voltage curves for the BTO (55 nm)/SiO_2 (285 nm) dielectric. (c) Dependence of the current *vs*. gate voltage curves as a function of the temperature. (d) Hysteresis of the current vs. gate voltage curves as a function of the temperature for the BTO_14 device.

Table S1. Summary of device characteristics for all devices measured. Values are reported or both the forward and back sweeps considering the hysteresis present in the gate sweeps. The width and length columns pertain to the semiconducting channel dimensions and the thickness column refers to the thickness of the BTO or hBN dielectric flake. (SS = subthreshold swing). Note that for the mobility calculation the average width along the MoS_2 channel is used. * Devices fabricated by transferring the BTO dielectric with the deterministic Nitto tape transfer method described in the main text. ** Devices fabricated by transferring the BTO dielectric with the Nail-polish pick-up and transfer method.

Name	ON/OFF Ratio	Hysteresis (V)	Forward Mobility (cm^2/V*s)	Back Mobility (cm^2/V*s)	Forward SS (V/dec)	Back SS (V/dec)	Average width (µm)	Min/max width (µm)	Length (µm)	Thickness (nm)
**BTO_1***	$2\cdot10^6$	24.0	4.7	7.9	2.3	0.7	7	6/8	25	47
**BTO_2***	$2\cdot10^5$	29.5	6.1	8.6	5.2	4.8	7	6.5/7.5	25	48
**BTO_7***	$5\cdot10^5$	16.0	22.2	31.1	1.6	1.9	7	6.5/7.5	25	45
**BTO_9***	$3\cdot10^7$	35.0	35.1	18.0	0.5	0.7	29	18/40	25	48
**BTO_10****	$6\cdot10^0$	44.0	56.7	52.4	26.6	351.1	10	7/13	30	50
**BTO_12****	$2\cdot10^0$	30.0	69.8	27.7	34.4	2678	19	11/28	25	~50
**BTO_14***	$1\cdot10^8$	21.0	49.5	47.8	1.2	1.4	30	25/35	25	55
**SiO_2_1**	10^6	4.5	9.9	10.3	0.4	0.6	17.0	17/17	30.0	--
**hBN_3**	$4\cdot10^{10}$	10.0	4.1	6.3	1.9	2.1	8.0	8/8	27.0	40.0
**hBN_8**	10^9	13.5	52.3	81.1	1.4	1.2	22	20/24	25.0	42.0
**hBN_10**	$2\cdot10^5$	33.0	1.5	0.9	1.4	1.9	15	11/20	25.0	34.0
**hBN_11**	$2\cdot10^8$	7.0	30.7	29.9	1.0	1.3	12	11/13	30.0	28.0

Supporting Information References:

[S1] Wemple, S. H.; Didomenico, M.; Camlibel, I. Dielectric and Optical Properties of Melt-Grown BaTiO3. *Journal of Physics and Chemistry of Solids* **1968**, *29* (10), 1797–1803. https://doi.org/10.1016/0022-3697(68)90164-9.

[S2] Mak, K. F.; He, K.; Lee, C.; Lee, G. H.; Hone, J.; Heinz, T. F.; Shan, J. Tightly Bound Trions in Monolayer MoS2. *Nature materials* **2013**, *12* (3), 207–211. https://doi.org/10.1038/nmat3505.

[S3] Baeumer, C., Rogers, S.P., Xu, R., Martin, L.W. and Shim, M. Tunable carrier type and density in graphene/PbZr0. 2Ti0. 8O3 hybrid structures through ferroelectric switching. *Nano letters*, **2013**, *13*(4), pp.1693-1698. https://doi.org/10.1021/nl4002052.

[S4] Lipatov, A., Sharma, P., Gruverman, A. and Sinitskii, A. Optoelectrical Molybdenum Disulfide (MoS2) Ferroelectric Memories. *ACS nano*, **2015**, *9*(8), pp.8089-8098. https://doi.org/10.1021/acsnano.5b02078.
